# Impact of Land-Use Intensity and Productivity on Bryophyte Diversity in Agricultural Grasslands

**DOI:** 10.1371/journal.pone.0051520

**Published:** 2012-12-12

**Authors:** Jörg Müller, Valentin H. Klaus, Till Kleinebecker, Daniel Prati, Norbert Hölzel, Markus Fischer

**Affiliations:** 1 University of Potsdam, Institute of Biochemistry and Biology, Potsdam, Germany; 2 University of Münster, Institute of Landscape Ecology, Münster, Germany; 3 University of Bern, Institute of Plant Sciences, Bern, Switzerland; Institute of Botany, Czech Academy of Sciences, Czech Republic

## Abstract

While bryophytes greatly contribute to plant diversity of semi-natural grasslands, little is known about the relationships between land-use intensity, productivity, and bryophyte diversity in these habitats. We recorded vascular plant and bryophyte vegetation in 85 agricultural used grasslands in two regions in northern and central Germany and gathered information on land-use intensity. To assess grassland productivity, we harvested aboveground vascular plant biomass and analyzed nutrient concentrations of N, P, K, Ca and Mg. Further we calculated mean Ellenberg indicator values of vascular plant vegetation. We tested for effects of land-use intensity and productivity on total bryophyte species richness and on the species richness of acrocarpous (small & erect) and pleurocarpous (creeping, including liverworts) growth forms separately. Bryophyte species were found in almost all studied grasslands, but species richness differed considerably between study regions in northern Germany (2.8 species per 16 m^2^) and central Germany (6.4 species per 16 m^2^) due environmental differences as well as land-use history. Increased fertilizer application, coinciding with high mowing frequency, reduced bryophyte species richness significantly. Accordingly, productivity estimates such as plant biomass and nitrogen concentration were strongly negatively related to bryophyte species richness, although productivity decreased only pleurocarpous species. Ellenberg indicator values for nutrients proved to be useful indicators of species richness and productivity. In conclusion, bryophyte composition was strongly dependent on productivity, with smaller bryophytes that were likely negatively affected by greater competition for light. Intensive land-use, however, can also indirectly decrease bryophyte species richness by promoting grassland productivity. Thus, increasing productivity is likely to cause a loss of bryophyte species and a decrease in species diversity.

## Introduction

Conservation of biodiversity is one of major ecological challenges nowadays [1]. In Central Europe, semi-natural grasslands are hotspots of biodiversity for both plants and animals [2]–[4]. However, these ecosystems severely declined in quantity and quality due to land-use change and intensification over the last few decades [5].

In semi-natural grassland ecosystems, investigations on relationships between land use and plant species diversity have only seldom considered bryophyte diversity [6]. Little is known about cryptogams such as bryophytes, although they are typical elements of grassland communities and conduce to fundamental ecosystem functions and processes such as carbon fixation [7]–[8] and the regulation of soil humidity and water retention capacity [9]. In addition, bryophytes can significantly promote or hamper germination and seedling establishment of vascular plants [10]–[12]. Furthermore, many bryophyte species are sensitive to environmental changes such as enrichment of nutrients, pollutants, or changes in humidity, and can thus serve as suitable ecological indicators for specific environmental conditions [13]–[16]. Similarly to vascular plants, bryophyte species can be affected by land use, either directly by mechanical impacts such as grazing and mowing, by toxic impacts of high nitrogen applications [17] or indirectly through increased productivity leading to asymmetric light competition with tall-growing plant species [4]. However, despite their relevance only few ecological studies in grasslands included bryophytes. These investigations were usually restricted to specific habitats such as fens [18]–[20], mountain grasslands [21]–[22], and dry calcareous grasslands [4], [23]–[27] or relied on artificial field experiments [15]. Bryophyte vegetation of rather common ecosystems like permanent agricultural grasslands was rarely studied or exhibited a very restricted species spectrum [13], [15].

We investigated the diversity of bryophytes in 85 agricultural grasslands in two different regions in Germany. On these grasslands we assessed relationships between bryophytes, land-use intensity, and grassland productivity. We included aboveground vascular plant biomass, nutrient concentrations therein and mean Ellenberg indicator values for vascular plants to assess environmental and productivity-related impacts on bryophyte diversity [13], [28], [29]. We further distinguished between acrocarpous (erect and usually small, with sporophytes on the top of the branches) and pleurocarpous species (creeping, with sporophytes on lateral branches) growth forms to test whether they respond differently to land use and productivity as they represent different ecological gilds. Pleurocarpous species occupy usually larger patches and are more persistent than acrocarpous species. Many acrocarpous species are fast colonizing or ruderal bryophytes and can rapidly increase after on soil disturbances.

Specifically, we addressed the following questions: i) Which major gradients of land-use intensity (fertilizer application, mowing and grazing intensity) affect occurrence and composition of bryophyte vegetation in agricultural grasslands? and ii) Which measures of productivity – biomass production, biomass nutrient concentrations, or Ellenberg indicator values – are most strongly related to bryophyte species richness? And third, as the most important question we ask: iii) How is the relationship between bryophyte vegetation and productivity in agricultural grasslands?

## Materials and Methods

### Study Area and Land-use Intensity

The study involved 85 grassland plots of 50×50 m size within two regions belonging to the setup of the long-term and interdisciplinary project of the Biodiversity Exploratories [30]: i) the UNESCO Biosphere Reserve Schorfheide-Chorin in the Northeast and ii) Hainich-Dün consisting of the National Park “Hainich” with surroundings in Central Germany. In Hainich-Dün all plots were on calcareous mineral soils with large clay content (Cambisols and Stagnosols), whereas in Schorfheide-Chorin in addition to sandy mineral soils (Cambisols, Luvisols, and Gleysols) on the glacial moraines, about half of the plots occurred on drained organic fen soils (Histolols), which are frequently flooded in winter and early spring. All grasslands are of seminatural origin and regularly used as meadows, pastures or mown pastures. Most common grass species were *Poa pratensis, Dactylis glomerata* and *Bromus hordeaceus.* Among the herbs *Taraxacum officinale, Cerastium holosteoides* and *Trifolium repens* belong to the most frequent species. Plots were selected in a randomly stratified manner from a larger pool of 500 study plots per region to represent a wide gradient of land use typical for central European agricultural grasslands. Information on land use was inferred from standardized interviews with farmers containing detailed information on management practices of the last three years 2006–2008. For each plot we calculated mean intensities of grazing (number of livestock units × days grazing × ha^−1^), fertilizer application (kg N × year^−1^ × ha^−1^) and mowing (number of cuts × ha^−1^ × year^−1^) as given in Blüthgen et al. (2012).

### Vegetation Survey and Biomass Analyses

Following the nomenclature of Koperski et al. [31] we recorded bryophyte species richness and estimated total bryophyte cover in a 4×4 m subplot in all 85 plots (Hainich-Dün: *n* = 43; Schorfheide-Chorin: *n* = 42) in April 2009, the season when bryophytes are most easily recognized. Bryophyte species richness was further separated into acrocarpous and pleurocarpous species (including liverworts) according to Hill et al. [14].

From mid-May to mid-June, vascular plants were recorded at the same plots. Based on these vegetation relevés, we calculated mean Ellenberg indicator values for nutrients, reaction, light, and moisture per plot without cover weighting [13]. As estimation variable for productivity, aboveground biomass of vascular plants was harvested after recording on each plot simultaneously by cutting the vegetation 2 cm above ground on 1 m^2^ as mixed samples of four randomly placed quadrates of 0.25 m^2^. Occasionally occurring shrubs and litter were excluded from the biomass sampling. Temporary fences prevented our plots from mowing and grazing before sampling took place. Biomass samples were dried for 48 h at 80°C, weighed, and ground to pass a 0.5-mm screen. Total nitrogen (N) concentrations were determined with an elemental auto analyzer (NA 1500, Carlo Erba, Milan, Italy). For the analyses of phosphorus (P), potassium (K), calcium (Ca) and magnesium (Mg) samples were digested in a microwave oven system (MLS Start, Milestone, Bergamo, Italy) with concentrated nitric acid (65%) and hydrogen peroxide (30%) and analyzed by ICP-OES (Vista-PRO Axial, Varian, Palo Alto, USA). All analyses were run in duplicates and repeated if results differed by more than 10%.

### Statistical Analyses

Canonical correspondence analysis (CCA) based on presence-absence data was used to assess patterns and gradients in the composition of bryophyte vegetation and to relate those to significant environmental factors (total inertia 2.74). Ordination was carried out with 83 relevés including all bryophyte species that occurred more than once in the dataset (30 species) and additional down-weighting of rare species. Furthermore, we used bi-plot scaling to optimize ordination for species data. All involved environmental variables (Ellenberg indicator values from vascular plants, vascular plant standing biomass, nutrient concentrations in biomass, nutrient ratios in biomass and the land-use intensity index) were standardized prior analysis (z-transformation) and forward selection with Monte Carlo permutation test (499 runs) was performed to assess the significance of environmental factors (p<0.05). Further, inflation factors of significant variables were checked for co-linearity. Ordination was performed using Canocoo 4.5 [32]. The entire list of bryophyte species used for CCA ordination is given in Table S1.

Afterwards, we calculated Spearman rank correlations between axes scores and environmental variables.

Because of partly correlated proxy variables for land-use intensity and productivity (biomass production and nutrient content therein and Ellenberg indicator values), we calculated three separate multiple regression analyses as linear models to test for their effects on bryophyte diversity. Prior to linear model calculations, species richness data were square-root transformed to achieve normal distribution. All statistical tests were carried out with JMP (JMP 5.1, SAS institute, Cary, North Carolina, USA).

## Results

### Bryophyte Species Richness and Cover

**Figure 1:**
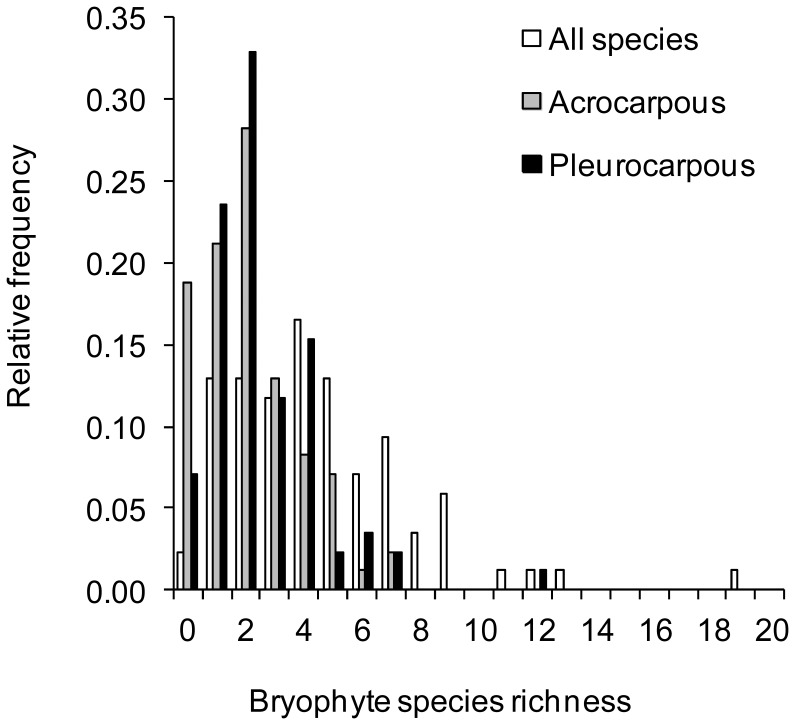
Frequency distribution of bryophyte species. Relative frequency distribution of the species richness of all, pleurocarpous, and acrocarpous bryophyte species per 16 m^2^ plots in grasslands (*n* = 85).

**Figure 2:**
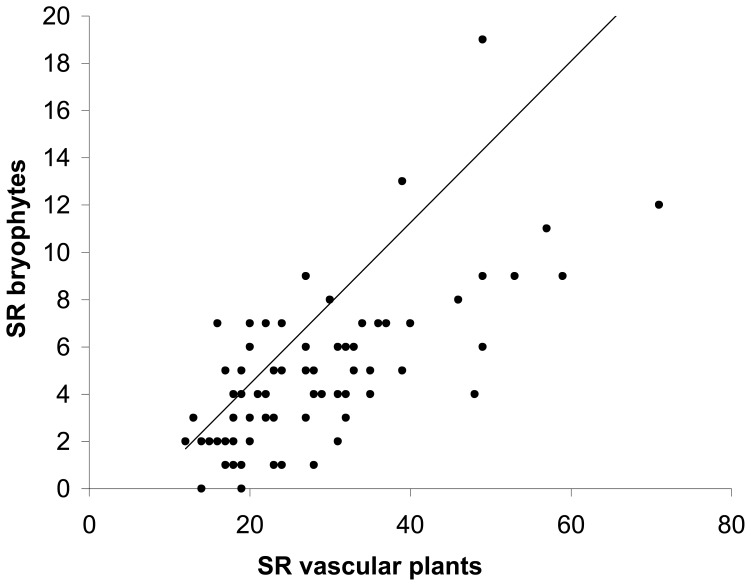
Relationship between species richness of bryophytes and vascular plants. Relationship between species richness of bryophytes and vascular plants (*n* = 85; y = 0.17x - 0.15; R^2^ = 0.47; F = 74.1; P<0.0001).

In total, we recorded 44 different bryophyte taxa on 84 of the 85 grassland plots (Table 1) constituting between 0 and 43% of total plant species richness on plots (mean: 16.6±0.95%). The most frequently occurring species were the pleurocarpous *Brachythecium rutabulum* (91% of all plots), *Eurhynchium hians* (57%), *Amblystegium serpens* (22%), and the acrocarpous *Phascum cuspidatum* (53%), *Barbula unguiculata* (28%), *Bryum rubens* (16%), and - only on drained fen soils - *Physcomitrium pyriforme* (16%). Generally, pleurocarpous species were more frequent than acrocarpous species (Fig. 1). In average we recorded 4.59±0.34 bryophyte species per 16 m plot but the species richness differed significantly among regions and were more than two times higher numbers in Hainich-Dün compared with Schorfheide-Chorin (Tables 1 and 2). This applied also for pleurocarpous and acrocarpous species separately. The total bryophyte species richness was positively correlated to species richness of vascular plants (Fig. 2). Mean bryophyte cover was relatively low in the studied grasslands (11.5±1.6%) and varied likewise among plots and study regions (Tables 1 and 2).

**Table 1:** Summary about bryophyte, land-use intensity, productivity and indicator value data.

	Hainich-Dün				Schorfheide-Chorin			
	Min	Max	Mean	±SE	Min	Max	Mean	±SE
Total species richness	2	19	6.4	0.5^a^	0	9	2.8	0.3^b^
Acrocarpous bryophytes	0	7	2.8	0.3^a^	0	5	1.3	0.2^b^
Pleurocarpous bryophytes	1	12	3.5	0.3^a^	0	4	1.4	0.1^b^
Cover of bryophytes [%]	3	60	21.5	2.2^a^	0	7	1.3	0.3^b^
Vascular plant species	16	71	33.8	1.8^a^	12	31	19.9	0.6^b^
Fertilization intensity [kg N*ha^−1^*year^−1^]	0	140	24.9	5.2	0	163	21.8	6.1
Mowing intensity [Cuts*year^−1^]	0	3.00	0.7	0.1	0	3	1.0	0.1
Grazing intensity [GVE*days^−1^ha*year^−1^]	0	1201	125	22	0	1362	110	27
Biomass [g/m^2^]	95.7	599.5	295.8	19.3	109.3	699.3	340.3	20.8
Ca [g*kg^−1^]	3.1	11.6	7.2	0.3	2.2	20.7	6.9	0.5
K [g*kg^−1^]	15.4	37.1	25.1	0.7^a^	4.5	30.4	16.2	1.0^b^
Mg [g*kg^−1^]	0.8	2.5	1.4	0.1^b^	0.7	5.6	2.1	0.2^a^
P [g*kg^−1^]	1.3	3.7	2.6	0.1	1.6	3.9	2.6	0.1
N [g*kg^−1^]	12.4	24.9	17.3	0.5^b^	8.6	35.2	19.9	1.0^a^
Ellenberg nutrient values	4.03	6.79	5.62	0.10^b^	4.82	7.38	6.11	0.08^a^
Ellenberg moisture values	4.29	5.69	4.99	0.05^b^	4.58	6.78	5.58	0.09^a^
Ellenberg light values	6.69	7.26	6.95	0.02^a^	6.60	7.23	6.86	0.02^b^
Ellenberg reaction values	6.00	7.20	6.69	0.04^a^	5.25	7.00	6.38	0.07^b^

Mean, minimum, maximum and SE of bryophyte diversity, vascular plant species, land-use measures, aboveground vascular plant biomass, nutrient content of biomass (Ca = calcium, K = potassium; Mg = magnesium, P = phosphorus; N = nitrogen), and mean Ellenberg indicator values for vascular plants for the two regions. Letters (^a, b^) indicate significant group differences between the regions.

**Table 2:** Summaries of multiple regression analyses.

Model A					
Source	*df*	Acrocarpous	Pleurocarpous	All species	Cover
Region	1	**23.32** ***	**56.18** ***	**52.18** ***	**273.59** ***
Fertilizer application	1	0.01	**15.27** **	2.94	0.59
Mowing intensity	1	**5.44** *	2.75	**6.00** *	0.10
Grazing intensity	1	0.36	0.64	0.01	1.89
Region × Fertilization	1	**4.27** *	3.27	0.55	0.01
Region × Mowing intensity	1	0.74	0.14	0.30	0.10
Region × Grazing intensity	1	0.31	0.43	0.74	3.40
Residual mean square	77	0.40	0.21	0.34	0.38
R adj.		0.25	0.46	0.40	0.77
**Model B**					
**Source**	***df***	**Acrocarpous**	**Pleurocarpous**	**All species**	**Cover**
Region	1	**20.74** ***	**60.02** ***	**56.41** ***	**293.1** ***
Biomass	1	0.32	**12.48** **	**6.04** *	3.36
Ca	1	0.15	**5.97** *	**4.02** *	0.84
K	1	0.50	1.74	0.75	0.37
Mg	1	1.32	**4.98** *	4.53	2.37
P	1	0.72	1.64	1.11	0.05
N	1	0.8	**6.29** *	3.78	**4.99** *
Residual mean square	76	0.45	0.19	0.31	0.35
R adj.		0.16	0.51	0.45	0.78
**Model C**					
**Source**	***df***	**Acrocarpous**	**Pleurocarpous**	**All species**	**Cover**
Region	1	**25.26** ***	**86.99** ***	**86.36** ***	**288.59** ***
Ellenberg nutrient values	1	**9.33** **	**69.0** ***	**58.72** ***	3.49
Ellenberg moisture values	1	**4.28** *	**5.12** *	**6.03** *	2.95
Ellenberg light values	1	1.14	0.83	0.03	2.19
Ellenberg reaction values	1	1.7	0.12	1.12	0.02
Residual mean square	79	0.37	0.14	0.21	0.36
R adj.		0.3	0.65	0.64	0.78

Summaries of multiple regressions of species richness of acrocarpous, pleurocarpous and all bryophytes as well as bryophyte cover on intensities of land-use procedures (Model A); aboveground biomass and their nutrient content (Model B), and mean Ellenberg indicator values for vascular plants (Model C) in the two regions. Levels of significance:

*** = p<0.0001;

** = 0.0001<p<0.01,

* = 0.01<p<0.05.

Further details concerning single variables are given in Table 1.

**Figure 3:**
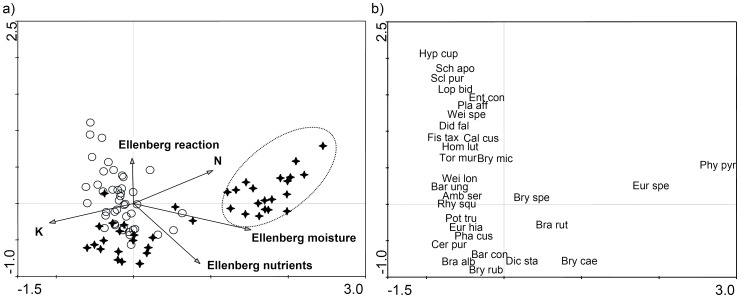
CCA-ordination of bryophyte species richness and environmental variables for stands (a) and species (b). CCA-ordination of bryophyte species and environmental factors in grasslands (n = 83). Stands and environmental factors (a) and species (b) of the same ordination are plotted separately to ease readability. Hainich-Dün: open circles; Schorfheide-Chorin: stars. Plots in the dotted circle are situated on either Gleyosols or Histisols. N and K are nutrient concentrations in aboveground vascular plant biomass and Ellenberg indicator values given are gained from vascular plant vegetation. Longer vectors indicate stronger correlations between variables and axes. See Table 1 for further details on single variables.

The CCA-ordination underlined significant differences in bryophyte composition between but also within study regions by widely separating some grassland plots along the first axis (15.3% explained variance) but also along the second axis (7.0% explained variance; Figure 3, Table S1). Due to strongly declining information content on the third and fourth axis (3.0 and 0.7%) we excluded them from further interpretation.

The first axis represents a gradient in especially moisture conditions (Ellenberg M indicator values, λ = 0.34, F = 11.32) but also in nutrient supply as revealed by Ellenberg N indicator values (λ = 0.06, F = 2.40) and N (λ = 0.10, F = 3.57) and K concentrations (λ = 0.14, F = 5.28) in vascular plant biomass (Table 3). These environmental factors widely separate some of the Schorfheide-Chorin plots from the rest of the plots. Only two bryophyte species, *Physcomitrium pyriforme* and *Eurhynchium speciosum*, had their main occurrence in these distinct plots in Schorfheide-Chorin, which are all situated on either Gleysols or Histosols, in contrast to all other species which either occur in both regions or mainly in plots in Hainich-Dün exhibiting less strong soil moisture (Fig. 3). The second ordination axis is characterized by soil reaction and arranges the plots assumingly along a gradient of soil pH.

**Table 3:** Spearman correlations of CCA-axes and environmental variables.

	Axis 1	Axis 2
Total species richness	−0.68**	–
Acrocarpous bryophytes	−0.51**	–
Pleurocarpous bryophytes	−0.71**	–
Cover of bryophytes	−0.52**	–
Fertilizer application	–	–
Mowing intensity	0.43**	–
Grazing intensity	−0.39**	−0.32**
Biomass	0.22*	−0.37**
Ca	–	0.57**
**K**	−0.58**	−0.27*
Mg	0.47**	0.23*
P	–	–
**N**	0.54**	0.47**
**Ellenberg nutrient values**	0.68**	−0.33**
**Ellenberg moisture values**	0.88**	–
Ellenberg light values	−0.41**	0.28*
**Ellenberg reaction values**	–	0.52**

Spearman correlations of CCA-axes and environmental variables (n = 83). Only significant correlations are shown. Levels of significance:

** = 0.0001<p<0.01,

* = 0.01<p<0.05.

Factors included in CCA factors are given in bold. Further details concerning single variables are given in Table 1.

### Land-use Effects

Bryophyte vegetation was negatively affected by certain land-use measures (Table 2). While total and pleurocarpous species richness were negatively related to cutting frequency, pleurocarpous species richness was reduced by high levels of fertilizer application. Additionally, acrocarpous species richness exhibited a significant negative relationship to fertilizer application but only in the Hainich-Dün region. In contrast, grazing intensity revealed no significant effects on bryophyte species richness. Bryophyte cover was unrelated to all land-use measures under study (Table 2). In summary, bryophyte vegetation was significantly influenced by land-use intensity, but less strongly compared with regional differences.

### Productivity Effects

**Figure 4:**
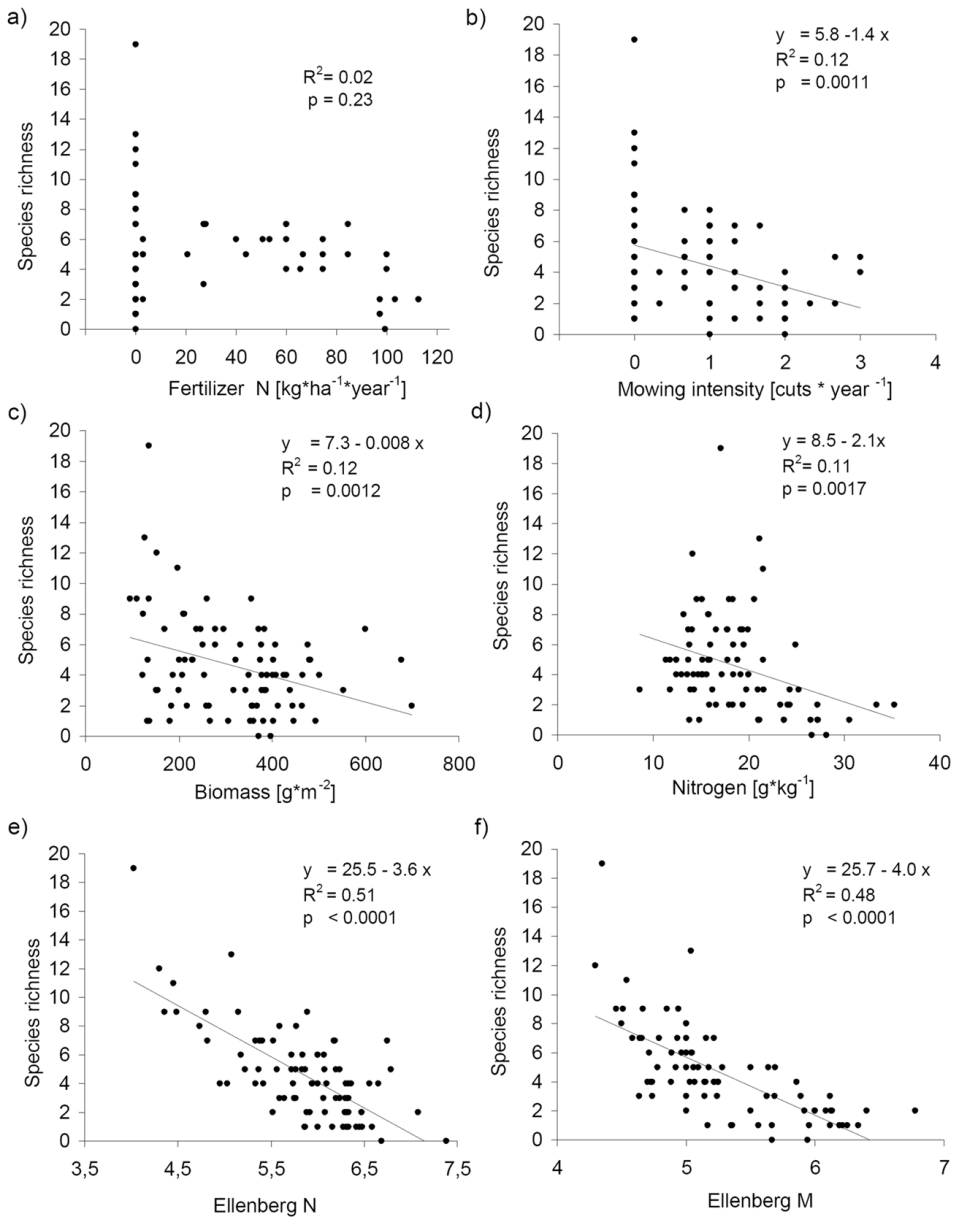
Relationships between bryophyte species richness and intensities of land-use and productivity. Relationships between total bryophyte species richness and intensities of land-use practices, mean annual amount of N fertilizer [kg*ha^−1^*year^−1^]; mean annual number of cuts [Cuts*year^−1^] from 2006–2008, aboveground community biomass of vascular plants [g*m^−2^], Nitrogen concentrations in biomass [[g/kg^−1^]], mean Ellenberg indicator values for vascular plants for nutrients (N) and for moisture (M) on 85 grassland plots.

Productivity affected total and pleurocarpous, but only to some extent acrocarpous bryophyte species richness (Table 2). Relationships between different measures of productivity and species richness varied significantly among variables under consideration. Both pleurocarpous and total species richness were significantly negatively related to biomass production (Fig. 4). Only Ca concentrations in vascular plant biomass were related to total bryophyte species richness, while for pleurocarpous species richness N and Mg concentrations were also significant (Table 2; Fig. 4). Acrocarpous species were neither affected by biomass production nor by any of the measured nutrient concentrations in biomass (Table 2; Fig. 4). Similarly, bryophyte cover was not related to biomass production, but exhibited a negative relation to N concentrations in biomass (Table 2). Potassium concentrations were not significantly related to bryophyte species richness in regressions, but in ordination analysis (Table 3). Therefore, we explored relationships between K and total species richness in each region separately. We found a significant negative relationship in Hainich-Dün (*r_S_* = −0.38; *p*<0.05) and a positive in Schorfheide-Chorin (*r_S_* = 0.37; *p*<0.05). As further implied by ordination analysis (Fig. 2), mean Ellenberg indicator values for nutrients and moisture were strongly negatively associated with total, pleurocarpous and acrocarpous species richness (Fig. 4). Thus, Ellenberg indicator values explained much more variation in total species richness than more direct measures of productivity, such as biomass and nutrient concentrations.

## Discussion

### Bryophyte Vegetation in Agricultural Grasslands

Bryophyte species were present in the vegetation of nearly all investigated grasslands, demonstrating that bryophyte vegetation can significantly contribute to plant diversity in agricultural grasslands. However, strong differences in species richness, composition and cover between study regions were observed, underlining the necessity of regional replications in ecological studies before the generalization of findings is reliable [33].

Species richness in Schorfheide-Chorin, where half of the plots are situated on drained fen soils, was significantly lower compared to the second study region Hainich-Dün. High soil moisture turned out to significantly reduce bryophyte species richness in agricultural grasslands (Fig. 4). This decrease especially in drained fen grasslands directly depends on the detrimental impact of strong seasonal fluctuations of the water table, which suppress both species typical for wet and species typical for dry habitats [34]. Flooding events during winter are typical for the lowlands in this region. These suppress the colonization of all xeric and most mesic bryophyte species, while soil desiccation during summer due to drainage prevented the establishment of species-rich bryophyte communities that are rather typical of calcareous fens [18], [35]. Under such conditions, vascular plants can acquire water from deeper levels due to their deep-reaching root system, outcompeting almost all small growing bryophytes on organic soils [36]. Additionally sandy soils on mineral sites cause faster desiccation and more imbalanced moisture dynamics on topsoil than loamy soils resulting in unsuitable conditions for bryophytes especially in precipitation poor regions like Schorfheide-Chorin. These moisture effects led to huge differences in total, acrocarpous as well as pleurocarpous bryophyte species richness between study regions. In addition to this we detected further effects of Ellenberg indicator values for reaction on bryophyte composition and species richness, illustrating the importance of soil conditions such as pH value for bryophyte vegetation. Furthermore, other factors on the regional scale such as land-use history may have also affected bryophyte species richness in this region, as reported for vascular plants [33]. Considering the low abundance of bryophytes in Schorfheide-Chorin, it became obvious that under these conditions bryophytes are prevented from fulfilling their ecological function to, for example, store nutrients during winter and as protective layer against surface wash off [37].

### Land-use Intensity

In contrast to studies on vascular plant diversity in grasslands, land use and in particular grazing intensity were only weakly related to all kinds of bryophyte species richness and cover [13], [38], [39]. However, fertilizer application and, closely correlated with this [40], mowing intensity significantly decreased bryophyte species richness assumingly due to intolerance of high nitrogen loads of most bryophyte species [14]. Fertilizer application stimulates growth of tall grasses and herbs, which results in enhanced light competition on the ground causing the exclusion of bryophytes [37]. Nevertheless, in our study, land-use intensity was by far less closely related to the composition of bryophyte vegetation in grasslands than expected. Possibly, effects of moisture and nutrient conditions due to peat mineralization and subsequent internal fertilization processes in drained fen soils might have partially overruled effects of fertilizer application, grazing and mowing intensity.

### Productivity Measures

Enhanced productivity had a strong detrimental influence on bryophyte species richness, clearly exceeding direct effects of land-use measures mentioned above (Table 2). Previous field studies and experiments stressed that increased nutrient levels fostering biomass production of vascular plants can be considerably harmful to bryophytes [36], [41]–[45]. However, to our knowledge, we confirmed this relationship for the first time across a broad range of permanent agricultural grasslands.

Although vascular plant biomass and N and Mg concentrations therein were negatively related to bryophyte species richness, mean Ellenberg indicator values for nutrients were most closely related to bryophyte species richness. Compared with point measurements, mean Ellenberg values are particularly suitable for the integration of variation in environmental factors in time and space [12], [46] and they are strongly related to biomass production [29]. Further, biomass estimation method might have lead to non-reliable results due to non-consideration of increasing re-growth ability of grasses after cutting or grazing in summer and variability due to different weather conditions among the two regions. Contradictory relationships between bryophyte species richness and K concentrations in biomass may result from different soil types among study regions. Contrary to grasslands on clay-rich mineral soils, grasslands on drained fen soils are often characterized by K deficiency due to high losses of K via leaching or removing of hay in intensive mowing regimes [7]. Along with enhanced N availability due to aerobic mineralization of peat, K deficiency causes higher Mg uptake by plants and therefore higher Mg concentrations in biomass [48]. Essentially, the enrichment of nitrogen in these soils favors highly competitive grasses and sedges, which effectively suppress bryophyte vegetation [33], [49]. Thus, K is positively associated with bryophyte species richness in Schorfheide-Chorin due to higher numbers of species in grasslands on mineral soils compared to those on drained fen soils. Meanwhile, highly productive grassland vegetation on clay-rich soils accumulates K when fertilized with this nutrient [15]. This has most likely led to a negative relationship between K concentrations in biomass and bryophyte species richness on all the mineral soils in Hainich-Dün. Against expectations, P concentration did not significantly affect bryophyte species richness, although P availability is an important driver of vascular plant species richness [38], [50]. Taken together, our results show that the Ellenberg indicator value for nutrients is the most useful and reliable proxy for environmental conditions which are detrimental to bryophyte vegetation in grasslands, irrespective of differences in soil conditions.

### Growth Forms

Among different growth forms, pleurocarpous bryophytes were more strongly affected by productivity than acrocarpous species. Due to their creeping growth form most pleurocarpous species have higher demands for light and relatively slow growth rates compared with acrocarpous species. Only few species such as *Brachythecium rutabulum* and *Eurhynchium hians* tolerate such dark conditions under dense herb layers. However, many acrocarpous species are also disadvantaged by highly productive conditions. Thus, generally bryophyte species were replaced by a couple of nitrophilous acrocarpous species such as *Phascum cuspidatum, Pottia truncata and* several *Bryum* species which are typical for arable land. These fast colonizing species depend on small disturbance patches such as wheel tracks, which are common in meadow swards.

### Conclusions

Bryophyte species richness in grasslands differed strongly between the regions especially due to differences in soil conditions and humidity. Moreover, in both regions our results demonstrate a strong negative impact of productivity and high nutrient levels on bryophyte vegetation in agricultural grasslands. Land-use intensity and in particular fertilizer application had negative effects on bryophytes, especially on pleurocarpous species. Nevertheless, moisture conditions of drained fen soils are assumed to have partially overruled relationships between land-use measures and species richness, at least in one region. Thus, site differences and indirect effects of land use such as drainage of fen soils were more important than direct measures of land-use intensity. However, both moisture and nutrient availability were strongly associated with each other. The mean Ellenberg indicator value for nutrients turned out to be the most powerful predictor to describe negative relationships between productivity and bryophyte species richness. Finally, our results underlined that land-use intensification to increase grassland yield is responsible for low diversity of bryophytes in agricultural landscapes.

## Supporting Information

Table S1 Abbreviations and full names of bryophyte species in NMDS ordination. Abbreviations and full names of bryophyte species in NMDS ordination displayed in Figure 2.
(DOC)
