# Supplementary material for: Impact of Land-Use Intensity and Productivity on Bryophyte Diversity in Agricultural Grasslands
Source: PLoS One. 2012 Dec 12;7(12):e51520. doi: 10.1371/journal.pone.0051520 (PMC3520803; doi:10.1371/journal.pone.0051520)
Supplement: Table S1 — Abbreviations and full names of bryophyte species in NMDS ordination. Abbreviations and full names of bryophyte species in NMDS ordination displayed in Figure 2. (DOC) [file pone.0051520.s001.doc]

**Table S1: Abbreviations and bryophyte species names in NMDS ordination.**

Abbreviations and full names of bryophyte species in NMDS ordination. (Figure 2).

| *Amb ser* | *Amblystegium serpens* |
| --- | --- |
| *Bar con* | *Barbula convoluta* |
| *Bar ung* | *Barbula unguiculata* |
| *Bra alb* | *Brachythecium albicans* |
| *Bra rut* | *Brachythecium rutabulum* |
| *Bry cae* | *Bryum caespiticium* |
| *Bry cap* | *Bryum capillare* |
| *Bry mic* | *Bryum microerythrocarpum* |
| *Bry rub* | *Bryum rubens* |
| *Bry spe* | *Bryum spec* |
| *Bry ten* | *Bryum tenuisetum* |
| *Cal cus* | *Calliergonella cuspidata* |
| *Cam cal* | *Campylium calcareum* |
| *Cam chr* | *Campylium chrysophyllum* |
| *Cer pur* | *Ceratodon purpureus* |
| *Cte mol* | *Ctenidium molluscum* |
| *Dic sta* | *Dicranella staphylina* |
| *Did fal* | *Didymodon fallax* |
| *Ent con* | *Entodon concinnus* |
| *Eur hia* | *Eurhynchium hians* |
| *Eur spe* | *Eurhynchium speciosum* |
| *Fis dub* | *Fissidens dubius* |
| *Fis inc* | *Fissidens incurvus* |
| *Fis tax* | *Fissidens taxifolius* |
| *Hom lut* | *Homalothecium lutescens* |
| *Hyl spl* | *Hylocomium splendens* |
| *Hyp cup* | *Hypnum cupressiforme* |
| *Lep pyr* | *Leptobryum pyriforme* |
| *Lop bid* | *Lophocolea bidentata* |
| *Pha cus* | *Phascum cuspidatum* |
| *Phy pyr* | *Physcomitrium pyriforme* |
| *Pla aff* | *Plagiomnium affine* |
| *Pla und* | *Plagiomnium undulatum* |
| *Ple sub* | *Pleuridium subulatum* |
| *Pot int* | *Pottia intermedia* |
| *Pot tru* | *Pottia truncata* |
| *Pte ova* | *Pterygoneurum ovatum* |
| *Rhy squ* | *Rhytidiadelphus squarrosus* |
| *Sch apo* | *Schistidium apocarpum* |
| *Scl pur* | *Scleropodium purum* |
| *Thu abi* | *Thudium abietinum* |
| *Thu phi* | *Thuidium philibertii* |
| *Tor mur* | *Tortula muralis* |
| *Wei lon* | *Weissia longifolia* |
| *Wei spe* | *Weissia spec* |
